# Understanding the episode of care for transplant patients

**DOI:** 10.1186/1472-6963-11-S1-A23

**Published:** 2011-10-19

**Authors:** P Weeks, I Blais

**Affiliations:** 1 MedAssets, Toronto, Ontario, Canada; 2 The Hospital for Sick Children, Toronto, Ontario, ON M5G 1X8, Canada

## Introduction

Funders and policy makers are looking for ways to manage increasing healthcare costs and provide funding for entire episodes of care that extend beyond a single stay or visit at a healthcare institution. In Ontario hospital funding is based on global funding, which is a lump sum distributed annually to hospitals. A small percentage of funding in Ontario is based on volume and fixed fees for selected procedures.

In a departure from the current funding policies, the Ontario Ministry of Health and Long-Term Care (MoHLTC) is formulating a strategy to increase patient based funding. This initiative is known as Patient based Payment (PbP). To execute this change in funding policy, the MoHLTC is extensively using patient level cost data to develop strategy and funding models. To gather evidence based information, the MoHTLC has funded a case-costing project to assist 48 facilities produce patient level cost data.

The current information systems and reporting standards in Canada are designed to record patient-care information by patient type during a single stay or visit in one institution. Although patients may receive health care from multiple healthcare providers, patient information is not linked or accessible by the multiple healthcare providers. The limitations of the current information systems have resulted in patient data that is difficult to access even within a single healthcare facility.

The single episode of care (one stay or visit) is not representative of the healthcare needed, or the entire cost of care related to a condition. This is particularly true for patients living with chronic diseases, or patients who have received complex medical procedures such as transplants and, ultimately, the healthcare services received throughout a patient’s life.

The transplant process is complex and can be divided into four phases: pre-transplant, assessment and waiting period; donor procurement; peri-operative; and post-discharge phase. Pre- and post-transplant healthcare services are not included in the procedure based funding and may be provided by multiple healthcare providers. Moreover, post-transplant care has increased as a result of improved survival rate resulting in continued healthcare.

In response, we propose to develop a linking of patient data describing complete care for all activities related to transplant. We suggest that a new model for episodic care is needed that provides funding for an entire episode of care. This will result in the proper incentives for improved care between providers. In this paper, we identify challenges and opportunities for this type of work in the future.

## Method

In 2008, The Hospital for Sick Children in Toronto (SickKids) examined the cost of transplant patients, including pre- and post-transplant healthcare services. This was done in order to understand the cost of the various organ transplantations throughout the full continuum of care. Using the SickKids transplant registry, 356 transplant patients were in this study. The study analyzed pre- and post-transplantation activity including inpatient admissions, outpatient activity (medical daycare and clinics), diagnostics, and allied health services (e.g. social work, physiotherapy, child life, dietetics). A full year of patient activity was linked to 44 transplantations encounters. The combined transplant and patient activity data was linked to the cost data.

## Result

The study demonstrated that the cost of transplantation for the peri-operative phase accounted for only 41.1% of the hospital’s total costs for treating transplantation patients. Pre- and post-inpatient activity accounted for 26.4% of the hospital’s costs, while ambulatory and diagnostic activity accounted for 32.5% of the hospital’s costs.

## Conclusion

Reporting systems currently in place limit the ability to describe resource intensity and clinical care for the full continuity of care. With the various complex and chronic patient populations that exist today, including transplants, there is an increasing demand on the healthcare system. Information systems need to be enhanced to link episodes in order to appropriately measure and fund patient activity and costs throughout a patient’s disease management.

The Hospital for Sick Children has extensive experience providing the continuum of care from initial diagnosis through to post-treatment care. Some of this care is delivered to patients over many years and in consultation and coordination with other healthcare providers. Yet despite this extensive clinical experience, the understanding of the delivery of care is difficult because of the disparate and silo-ed patient information. Funders, healthcare and information management providers should work together to pool knowledge and experiences and collaboratively design episode of care models. The episode of care model can be the catalyst for new approaches to delivering healthcare.

